# Distinguishing butchery cut marks from crocodile bite marks through machine learning methods

**DOI:** 10.1038/s41598-018-24071-1

**Published:** 2018-04-10

**Authors:** Manuel Domínguez-Rodrigo, Enrique Baquedano

**Affiliations:** 1 Institute of Evolution in Africa (IDEA), University of Alcalá de Henares, Covarrubias 36, 28010 Madrid, Spain; 2 Department of Prehistory, Complutense University, 28040 Madrid, Spain; 3 Regional Archaeology Museum, Alcalá de Henares, Plaza de las Bernardas s/n, 28801 Madrid, Spain

## Abstract

All models of evolution of human behaviour depend on the correct identification and interpretation of bone surface modifications (BSM) on archaeofaunal assemblages. Crucial evolutionary features, such as the origin of stone tool use, meat-eating, food-sharing, cooperation and sociality can only be addressed through confident identification and interpretation of BSM, and more specifically, cut marks. Recently, it has been argued that linear marks with the same properties as cut marks can be created by crocodiles, thereby questioning whether secure cut mark identifications can be made in the Early Pleistocene fossil record. Powerful classification methods based on multivariate statistics and machine learning (ML) algorithms have previously successfully discriminated cut marks from most other potentially confounding BSM. However, crocodile-made marks were marginal to or played no role in these comparative analyses. Here, for the first time, we apply state-of-the-art ML methods on crocodile linear BSM and experimental butchery cut marks, showing that the combination of multivariate taphonomy and ML methods provides accurate identification of BSM, including cut and crocodile bite marks. This enables empirically-supported hominin behavioural modelling, provided that these methods are applied to fossil assemblages.

## Introduction

In the early 1980s, the discovery of butchery marks on fossil bones from the oldest anthropogenic site (FLK *Zinj*, Olduvai Gorge) produced a series of analyses that led to the taphonomic confirmation of cut marks as linear grooves with V-shaped cross-sections and internal microstriations^1,2^. Subsequently, it was found that natural marks created through sediment abrasion and trampling could also mimic cut marks in cross-section shape and internal microstriations^3–5^. At the same time, it was discovered that stone-tool-imparted cut marks resulted in a diverse repertoire of cross-section shapes, well beyond the ideal V-shape section^6,7^. More recent work uncovered that bone surface modifications (BSM) with similar characteristics could result from the action of other agents, such as crocodiles^8^, vultures^9^, and other carnivores using their claws^10^. Since then, most taphonomists have abandoned the exclusive use of these two variables (V-shaped section and internal striae) to identify cut marks in the fossil record, because they lend themselves to equifinality.

**Figure 1:**
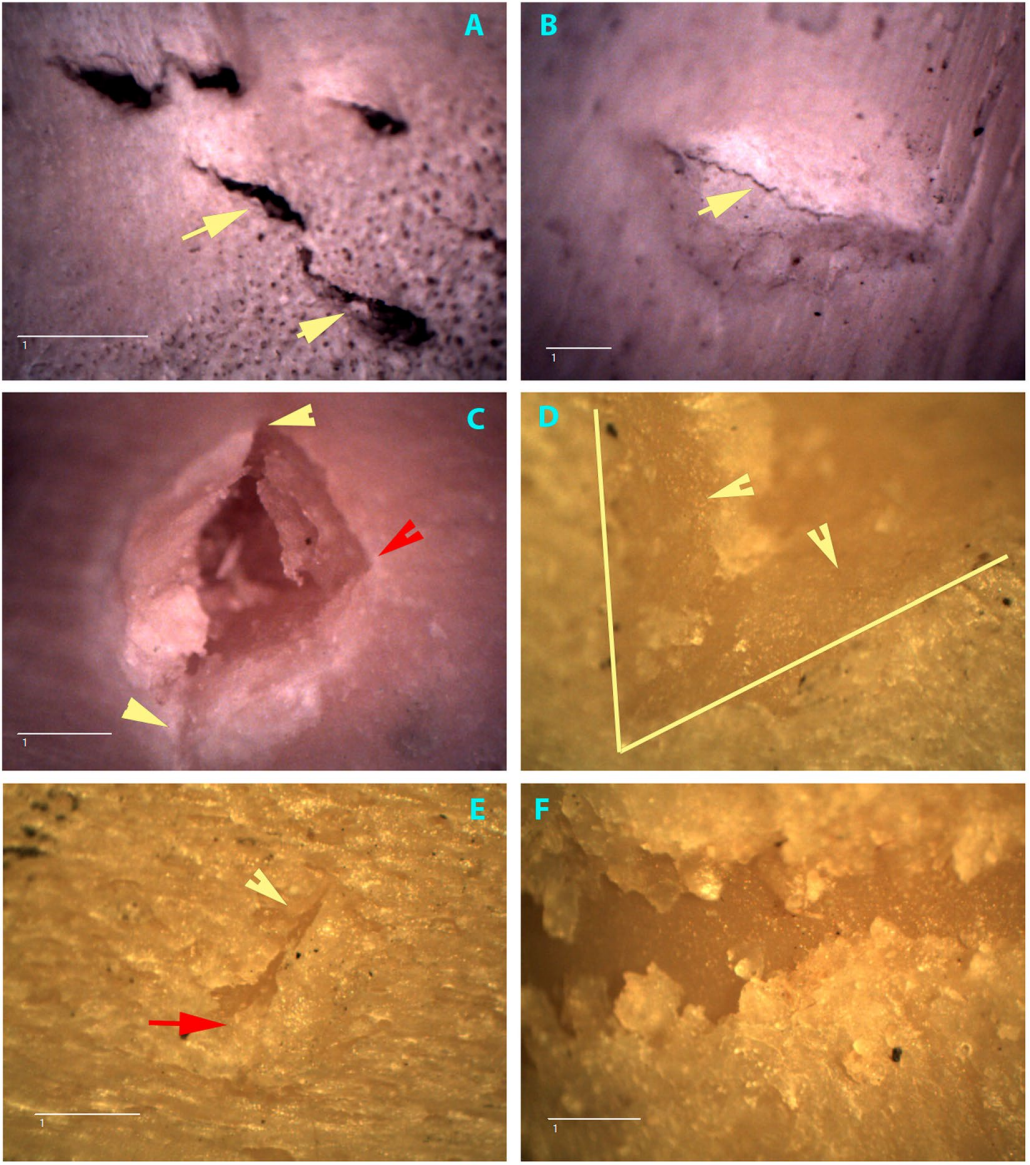
(**A**) Five elongated semi-punctures caused by crocodiles carinated teeth. Notice the irregular outline of the mark (arrows). This irregular outline can also be found frequently in the carinae of crocodile tooth pits (**B**, arrow). (**C**) Another pattern of crocodile tooth pit, showing slight protuding ends of the carinae (yellow arrows) and angular section on one side (red arrow). These types of “triangular marks are also common in BSM made with unworn crocodile teeth. (**D**) Section of a perfect V-shaped tooth score made by crocodiles. Notice de absence of internal striae on the walls of the mark. (**E**) Typical combination of tooth pit (red arrow) plus V-shaped linear groove (yellow arrow) very abundantly represented in crocodile BSM. (**F**) Classical U-shaped tooth score without internal striae made by crocodiles. This type of BSM is common and completely overlooked by several studies because of its lack of overlap with stone-tool cut marks. Images are at 20× . Scale = 1 mm.

**Figure 2:**
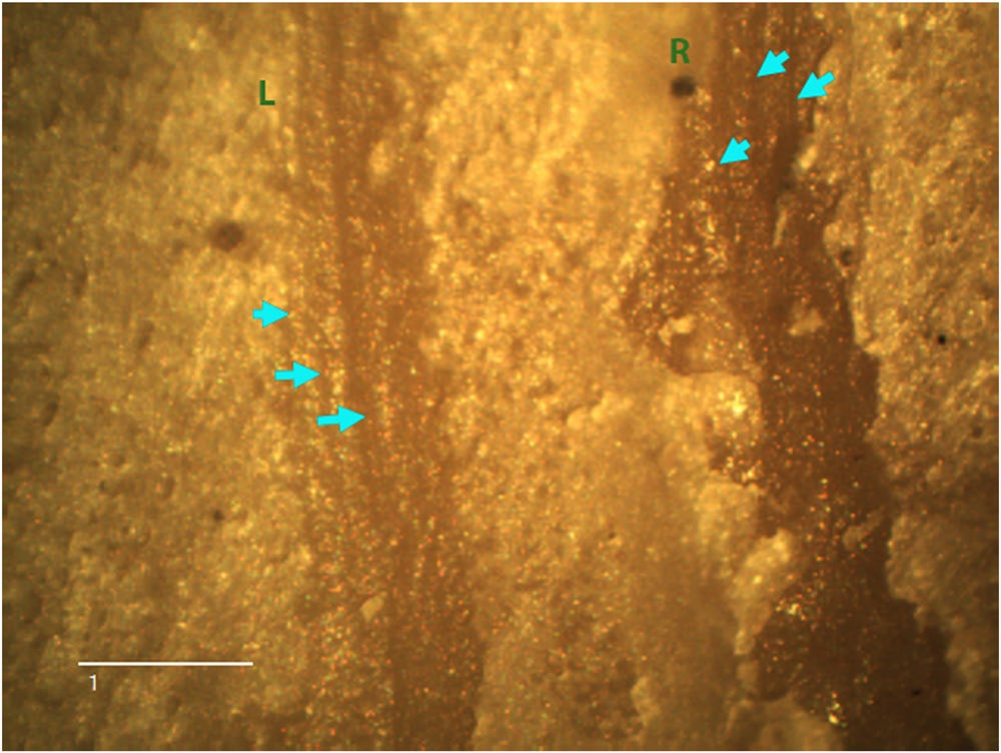
Good examples of linear tooth scores made by crocodiles in which internal microstriations are continuous (L) or mostly absent (R). In L, the striae are asymmetrically located on one side of the groove, with small number of striations and great separation in between them. This contrasts with the greater number of tightly packed microstriations found in stone-tool marks. In R, three striae, which follow the same pattern of separation are suddenly interrupted and most of the score is striae-free. Notice the difference in the flaking and shoulder of both marks. Only L is V-shaped. Image at 25× . Scale = 1 mm.

It is therefore surprising that a recent study questions the ability of taphonomists to ascribe agency to BSM, based on the already-known fact that crocodiles also make V-shaped and linear microstriated BSM^11^. This new study resurrects the outdated assumption based upon just the two variables mentioned above and it concludes, predictably, that equifinality prevents most V-shaped and linear microstriated BSM to be correctly attributed to agent. This study flies in the face of ample taphonomic evidence that multivariate approaches succeed where older uni- or bivariate approaches failed. Trampling and cut marks made with different stone tools were successfully identified under controlled experimentation in more than 90% of cases when more than a dozen variables of microscopic features of BSM were used simultaneously^12,13^. A similar multivariate protocol also enabled the differentiation of cut marks from trampling, hyena, and crocodile tooth marks at rates >80%^14^. Recently, machine learning (ML) methods on a sample of 1000 BSM have increased (depending on the algorithm type) the identification of marks to almost 100% accuracy^15^. Why then do Sahle *et al*.^11^ resurrect the same equifinality argument? Can crocodile and cut marks really be confused, impeding heuristically-supported discussions of early human behaviour and sending interpretations of Early Pleistocene sites and hominins to an epistemological limbo? (Figs 1 and 2)

Here, we use successful ML methods to compare trampling marks, cut marks made with stone tools (using simple and retouched flakes), and crocodile bite marks. These four types of BSM are formally similar (V-shaped to various degrees and with internal striae); however, they differ in several other aspects. A sample of 10,000 marks from controlled experimental sets are used to train and test the algorithms with the highest classification accuracy. The intrinsic and extrinsic information contained in each BSM and their context can be used to successfully discriminate most marks. This has serious repercussions for paleoanthropology, since most behavioural debates on early human evolution revolve directly or indirectly on unravelling information contained in BSM in archaeofaunal assemblages. The heuristics of interpretations made of any given site will thus depend upon which taphonomic methods have been used for correct BSM identification and interpretation.

## Results

A series of eight ML algorithms were used in the BSM sample, using a total of 17 variables (complete set) (Table S1). All of the algorithms correctly classify more than 96% of all marks (Table 1). Three algorithms are less efficient than the others: Naive Bayes (NB), Partial-Least Square Discriminant Analysis (PLSDA) and Mixture Discriminant Analysis (MDA). NB shows the lowest sensitivity of these three. In contrast, Neural Networks (NN), Support Vector Machines (SVM), K-Nearest Neighbour (KNN), Random Forests (RF) and Decision Trees using the C5.0 algorithm (DTC5.0) show maximum accuracy in the correct classification of all BSM, with perfect sensitivity (1.0) and specificity (1.0) in all BSM types. This shows that several ML techniques are able to correctly classify and discern BSM and crocodile bite marks from other similarly-shaped BSM at a rate of 100% accuracy (Table 1).

**Table 1:** Accuracy values for each algorithm/test. Kappa, sensitivity and specificity values are also included.

Complete set (intrinsic and extrinsic variables)					
	accuracy	95%CI	kappa	sensitivity*	specificity*
NN	100	0.99-1	1	(1.0,1.0,1.0,1.0)	(1.0,1.0,1.0,1.0)
SVM	100	0.99-1	1	(1.0,1.0,1.0,1.0)	(1.0,1.0,1.0,1.0)
KNN	100	0.99-1	1	(1.0,1.0,1.0,1.0)	(1.0,1.0,1.0,1.0)
NB	96,86	0.961-0.974	0,95	(0.59,1.0,1.0,0.98)	(1.0,0.99,0.96,0.99)
RF	100	0.99-1	1	(1.0,1.0,1.0,1.0)	(1.0,1.0,1.0,1.0)
PLS	96,73	0.960-0.973	0,95	(0.72,0.97,0.97,0.99)	(1.0, 0.99, 0.96,0.98)
MDA	99,33	0.989-0.995	0,99	(0.92,1.0,1.0,0.99)	(1.0,0.99,0.98,1.0)
C5.0	100	0.99-1	1	(1.0,1.0,1.0,1.0)	(1.0,1.0,1.0,1.0)
**Partial set (intrinsic variables)**					
NN	99,63	0.993-0.998	0,99	(1.0,1.0,1.0,0.98)	(1.0,0.99,1.0,1.0)
SVM	99,07	0.986-0.993	0,98	(0.94,1.0,1.0,0.98)	(1.0,0.99,0.99,1.0)
KNN	99,63	0.993-0.998	0,99	(1.0,1.0,1.0,0.98)	(1.0,0.99,1.0,1.0)
RF	99,64	0.993-0.998	0,99	(1.0,1.0,1.0,0.98)	(1.0,0.99,1.0,1.0)
C5.0	99,63	0.993-0.998	0,99	(1.0,1.0,1.0,0.98)	(1.0,0.99,1.0,1.0)

*(croc,rf,sf,tramp). Key: croc, crocodile tooth marks; rf, retouched flakes; sf, simple flakes; tramp, trampling.

It could be argued that some of those extrinsic (i.e., contextual) variables that have discriminatory potential do not take into account the palimpsestic nature of the fossil record by considering superimposition of BSM from different processes. Thus, variables such as “microabrasion” (which effectively contribute to discerning trampling marks from cut marks) or tooth pits associated with linear BSM (which effectively help discerning crocodile tooth scores from other BSM) could be less influential in other potential scenarios. That would be the case of trampling over cut-marked bones, or butchery of scavenged carnivore-consumed carcasses. For this reason, in order to assess BSM by their structural characteristics, extrinsic variables were removed in a second analysis, and only intrinsic (i.e., structural) variables were used (see definitions in Table S1). This was referred to as the partial set analysis.

The ML algorithms that successfully identified BSM in 100% of cases in the analysis of the complete variable set were selected for testing this partial variable set. In this case, despite the loss of information by removing variables, these algorithms correctly classified BSM in more than 99% of cases. NN, KNN, RF and DTC5.0 were the most accurate classifiers. In the results provided by these four algorithms, the few misclassifications affected trampling marks and cut marks made with retouched flakes. The four algorithms yielded a sensitivity of 1.0 and a specificity of 1.0 regarding crocodile BSM. This means that all these ML methods provided an accurate classification (100%) of all the crocodile marks and no other BSM was misclassified as a crocodile linear mark.

## Discussion

There are two traditions in taphonomy. One is anchored in the twentieth century and its focus is on registered taphonomic entities (i.e., objects) as individual entities, and on assemblages as concentrations of entities^16^. This additive concept of taphocenoses and ichnoenoses is individualistic. Regarding BSM, such an approach places special emphasis on quantification of taphonomic attributes of each mark separately. Methodologically, this approach to BSM uses taphonomic variables independently.

An alternative taphonomic approach, developed in the twenty-first century, is systemic (as opposed to individualistic) and emphasizes that association of registered taphonomic entities, via assemblage formation, entails emergent properties beyond the mere addition of the properties of single taphonomic objects. Taphonomic attributes are thus not just the ones pertaining to each single taphonomic entity, but also those emerging from associations of entities. This systemic approach has strong parallels with evolutionary biology^16^. Taphonomic entities are considered dynamic entities that occur in specific taphotopes, organized in the form of taphons (i.e., taphonomic groups) and they even occasionally display the property of being reproductive by creating new taphonomic entities^16–18^. Regarding BSM, emergent properties can only be addressed through multivariate approaches and this entails using not only all variables simultaneously, but also complex statistics. Each BSM contains its own structural information, in addition to that resulting from its association with other BSM on the same bone specimen as well as other features of the supporting bone specimen. This is what was defined as a configurational (i.e., relational) approach^19^. A configurational approach requires intrinsic (i.e. pertaining to the morphological and internal characteristics of a mark) and extrinsic (i.e., association of a specific mark with other marks or features on the same bone specimen) variables.

Here we have shown that multivariate configurational ML methods can successfully classify all experimental BSM, with an accuracy rate of 100%. This method uses a combination of intrinsic and extrinsic properties of each BSM, which shows that only configurational approaches capture the additional and emergent associative properties of marks on any given bone specimen. The important feature of this method is not only its high accuracy in mark identification, but also that it provides a computer-based way to classify BSM, avoiding the subjective classification made directly by the analyst. This does not remove subjectivity completely, but it reduces it significantly^15,20^. This work reinforces previous studies, which showed high success in BSM classification when using multiple variables^12,14^. It also shows that independent variables (such as cross-section shape and internal striae), may produce equifinality if considered separately, but may provide higher resolution if considered in conjunction with other variables. For example, V-shaped marks that also display intense flaking and abundant shoulder effects are much more frequent in cut marks made with retouched tools than in other BSM.

Sahle *et al*.^11^ subjectively classified BSM on Plio-Pleistocene bones from Ethiopia without any quantitative or qualitative support. These interpretations, based on descriptions, are not even based on a mark-by-mark comparison with modern experimentally controlled BSM, and no convincing matches were provided. These authors qualified statistically-based analyses as narrowly mark-focused and promising little illumination. This unfounded statement is contradicted by the present ML study. The present multivariate analysis sheds more light on BSM classification than the equifinal groove-shape section approach used by Sahle *et al*.^11^. These authors misrepresent taphonomic praxis when they depict current taphonomic work as “roadblocking knowledge” and as shaped “by a tool/carnivore dichotomy”. This dichotomy is truly non-existent. This perception may have been projected because some research teams focused for decades mostly on carnivore BSM to indirectly interpret hominin subsistence, but they do not represent the praxis of the paleoanthropological taphonomic community. Taphonomists are usually concerned with all types of BSM equally, because all contribute to information about site formation. The only obstacles to knowledge are epistemological and methodological, as the present work shows.

The “universe of equifinality” means that Sahle *et al*.^11^ also fail to consider alternatives to their interpretation of crocodile-modified bones from Middle Awash. If considering those marks in isolation using the striated cross-section shape, many of them cannot be differentiated from marks made by vultures^9^, trampling^12^, gravel compaction^21,22^ and even stone tools. These authors have not reproduced marks made by the diverse array of tools potentially creating BSM (e.g., modified cores). How are these different from the marks they report (see ref.^23^ for similarities)? They have not rejected these alternative agents, meaning that, epistemologically, they cannot support crocodile agency. If, on the contrary, they were trying to be “consilient” and were considering marks in their context, they could find better support for some of the specimens they present, given the conspicuous tooth mark patterns of crocodiles on bones. Both Njau^8^ and Baquedano *et al*.^24^ report that between 75% and 82% of crocodile tooth-marked elements bear carinated marks, typical of crocodiles, frequently among other abundant BSM (especially tooth pits) on the same specimen. The presence of diverse marks on the Middle Awash bones could support Sahle *et al*.’s inferences, although they do not report any frequency of carinated marks. This configurational approach also indicates that the FLK *Zinj* assemblage, fundamental for interpretations of early human behaviour, shows very low frequency of multiple non-human carnivore-imparted marks on the same specimen (most commonly just one), very low frequency of pits, and not a single carinated BSM in the assemblage, thereby refuting the hypothesis of crocodile modification of the bones accumulated at the site, and supporting predominantly hominin agency^25^, as reflected by the assemblage’s abundant unambiguous cut mark record.

Sahle *et al*.^11^ argue that there is no technological fix to the equifinality resulting from the application of sophisticated 3D techniques to BSM. This perception results from two factors. First, the authors´ disregard for statistical treatment of data leads to a failure to appreciate significant differences between their own butchery and crocodile mark data sets, which they obtained through BSM profile analysis (see our analysis of their data in Supplementary Information). In fact, their data show that none of the fossil specimens that they selected bear unambiguous crocodile BSM. In contrast, some of them cluster closely with their own butchery data (Supplementary Information). Second, the authors selected the least developed and least experimentally tested of the 3D techniques available, instead of selecting the multivariate geometric morphometric techniques tested in larger experimental samples. The latter have yielded positive results in discriminating tool types and raw material types through the analysis of cut marks^26–29^. This and other more sophisticated 3D techniques have also successfully discriminated among BSM caused by different mammalian carnivores^30^ and even distinguished accurately tooth marks made by crocodiles from those inflicted by other carnivores^31^. A key element in this success was that the techniques were statistically complex, and that the focus was not on isolated marks, but on the ichnological assemblage. This is another advantage of the systemic approach. In contrast, Sahle *et al*.’s^11^ interpretations of fossil BSM are based on single specimens, completely out of context or with no directly associated faunal assemblage, despite their assertion that heuristic interpretations can only be made at the assemblage level, not with isolated specimens or, even worse, isolated BSM.

The tested accuracy in differentiating cut marks from crocodile marks presented with data here, in contrast with the descriptions provided by Sahle *et al*.^11^, could also result from the pristine conditions of the controlled assemblages, where each of them represents a single process. Bones and their ichnological content are dynamic taphonomic entities subjected to multiple processes, each of them adding and deleting information. How, for example, could a cut-marked bone subjected to post-depositional processes such as sediment abrasion, modify the BSM associative properties? This cannot be answered because we lack experimental data on palimpsestic ichnological processes. For the moment, we can approach this question by removing the extrinsic variables from our analysis, as was done above. Even the intrinsic variables would be affected by superimposed processes^32^, but given the lack of experimentation, we cannot elaborate on that here either.

Crocodile marks were correctly classified among other things because of the high presence of other marks (especially pits) on the same specimen. If this extrinsic information (as well as other extrinsic variables) is removed, the ML methods can still classify correctly >99% of crocodile BSM. Cut marks made with simple flakes are also classified correctly in 100% of the tested samples.

The present work reinforces the idea that taphonomy (and palaeontology) can no longer be a descriptive discipline – as was the case during most of the twentieth century – in which descriptions are subject-dependent and variables are used independently. This work also emphasizes that systemic configurational approaches are heuristically stronger than individualistic context-free approaches. Replicable data must be generated and used in a multivariate manner. ML algorithms capture variable interaction like no other analytical tool. The application of this method to experimental marks shows that in general, most cut marks can be differentiated from crocodile bite marks. This does not necessarily mean that such accuracy can be achieved in the fossil record, but it certainly indicates that equifinality in fossil contexts can be heuristically overcome, because the probabilities of BSM classification will differ among different mark types. We have explicitly avoided getting lost in theoretical praxiological advice, such as that displayed by Sahle *et al*.^11^; rather, we have described in detail the methodological basis to overcome a purported, no longer existent equifinality in the identification of cut marks and crocodile bite marks.

## Method

The set of variables used for the present study is described in Table S1. The original BSM sample consisted of 105 cut marks made with retouched flakes, 246 cut marks made with simple flakes, 224 trampling marks and 58 tooth marks (scores) made by crocodiles. The original experimental BSM samples are described in refs^12,24^. In order to provide the modelling with large training and testing/validation sets, the sample was bootstrapped 10,000 times, yielding a sample that is substantially bigger than BSM samples that one may encounter in archaeofaunal assemblages. A total of 70% of this sample was used for the training models. Testing/validation was carried out on the remaining 30% of the sample. This is a standard procedure in predictive models in order to deal with the bias/variance tradeoff. In the present work, the sample was initially bootstrapped with a function from the “caret” R library that considers bootstrapping the sample in proportion to the variable representation to each of the factors of the outcome variable. Categorical variables were transformed into numerical (dummy) variables. After enlarging the sample, data were pre-processed. To minimize variance biases, data were centred and scaled. The ML algorithms used did not require data transformation to deal with normality, skewness or collinearity.

During the application of ML algorithms, the models were tuned with self-correcting methods. This is one of the great advantages of ML tests. During model elaboration, several techniques allow estimating the performance of the model. Some statistics (e.g., RSME) enabled estimating the performance potential on new data. Several and very diverse ML algorithms were compared for efficiency and accuracy. Model evaluation took place through resampling techniques that estimate performance by selecting subsamples of the original data and fitting them in multiple submodels. The results of these submodels were aggregated and averaged. Several techniques can be used for this subsampling and submodelling: generalized cross-validation, k-fold cross-validation, leave-one-out-cross validation or bootstrapping. Here, we selected 10-fold cross-validation, which consists of the original sample being partitioned into 10 similarly-sized sets. A first model is subsequently generated using all subsamples, except the first fold. Then the first subset is reintroduced to the training set and the procedure is repeated with the second fold and so on until the tenth one is reached. The estimates of performance of each of the ten processes are summarized and, thus, used to understand the model utility.

Once all models are completed, model selection takes place. his is usually done combining indicators of error (i.e., RSME or root mean square error) or accuracy. Cost values (of bias-variance) were evaluated vis-a-vis accuracy with the caret function “tuneLength” up to 10 (i.e., 2−^2^…. 2^7^). The tuning parameter selected for measuring model performance was the “kappa” parameter. For class predictions, these can come in two forms: a discrete category (showing the factor classification) and a probability of membership to any specific category. This latter can be continuous (as in random forests or discriminant analyses, for example) or binary when using sigmoid classifiers (as in logistic regression or support vector machines). The Kappa statistic (which considers the amount of accuracy generated by chance) can take the form of −1 to 1 (as in correlation). It is a proxy of accuracy by indicating a perfect match between the model and the documented classes (kappa = 1) or a less than perfect match (kappa = < 1). Kappa values of 0.3–0.6 shows reasonable agreement. Estimates higher than these indicate a high agreement between the expected accuracy and the documented one. Cohen´s kappa value is a more robust measure of prediction and classification than accuracy, because it does not quantify the level of agreement between different datasets, but it represents the degree of similarity of datasets corrected by chance. The selected model performance was also tabulated with confusion matrices.

Machine learning techniques are powerful predictive and classification methods^33^. A selection of those identified as the most powerful classificatory methods available^34^ were used and compared. These comprised the following (some of them initially described in ref.^35^):

### Neural Network (NN)

This algorithm operates by creating nodes which hierarchically build a network of synthesized information through regression methods. This works similarly to the neural networks of the human brain. Nodes convey the transformed input signal through feedforward networks, which terminate in an output node. The training of the neural network is done by adjusting weights through successive layers of nodes. The input data fed to the neural layers (perceptrons) is transformed via specific nonlinear sigmoidal functions. The parameters of these functions are usually optimized to minimize SQR (Sum of Square Residuals). These parameters exhibit a tendency to overfit the training data set. To avoid this, weight decay is used to reduce the model errors for a given value of lambda. This λ parameter must be specified together with the number of hidden units (perceptrons). Reasonable values for λ range from 0.0 to 0.1. Here, five different weight decay values were tested (0.00, 0.01, 0.1, 1, 2). The models were tuned for an uneven number of units (i.e., neurons) ranging from 1 to 19, in a resampling method involving training and testing subsamples. The final values for the complete set model were size = 5 and decay = 0.0177. The final values for the partial set model were size = 10 and decay = 0.0021. For the present analysis, the “nnet” and the “caret” R libraries were used.

### Support Vector Machines (SVM)

The SVM algorithms provide a powerful method for non-linear classification. A SVM is a mathematical and spatial boundary between data points in a multidimensional space. It creates a hyperplane which yields homogenous distribution of data on either side. In non-linear spaces, data separation is achieved through the use of kernels, which add additional dimensions to data in order to achieve a proficient separation according to class. The SVM regression method uses a threshold (via the tuning of kernels) set by the user to determine which residuals contribute to the regression fit. To estimate the model parameters, SVM also uses a loss function. The cost (C) parameter is the cost penalty that is used to penalize models with large residuals. The loss function (the same as the lambda in NN) determines the degree of overfit of the training data. The cost parameter adjusts the structure of the model. The algorithm used in the present study was the C-Classification parameter with a SVM radial kernel. The size of the hyperplane is selected through the value of C. Large values of C will produce a small-margin plane to maximize classification. Low C values produce a wider plane resulting in higher rates of misclassification. Here, a fixed value for the cost function was adopted and the kernel parameter was estimated to σ = 0.0521 for the complete set analysis and σ = 0.0671 for the partial set analysis. The model was tuned over >100 cost values. The final cost value selected by the kappa parameter was C = 4 for the complete set analysis and C = 128 for the partial set analysis. For the present study, the “e1071” and the “caret” R libraries were used.

### K-Nearest Neighbour (KNN)

This unsupervised (lazy) learning algorithm classifies unlabelled data by assigning them the class of the most similar labelled examples. This algorithm works well in samples with many variables and performs well when there are well-defined labelled sets. The algorithm makes no assumption about the distribution of the sample and it is easy to train. KNN identifies *k* cases in the sample as the nearest in similarity. Unlabelled cases are subsequently assigned by similarity. To predict the location of testing data in the predictor space, different k models are tested and compared to an error/accuracy parameter. To overcome the bias-variance tradeoff an intermediate *k* value is usually selected. Larger k values tend to reduce the bias of variance but small patterns may go unnoticed. Here, a final model was produced with k = 5 (both for the complete set and partial set analyses), after having tested 32 and 23 different *k* values respectively. Training and testing datasets were created through boosted subsamples. These were subsequently analysed using the R “class” and “caret” libraries and the “knn” function.

### Random Forest (RF)

The algorithm uses a small random number of the dataset variables, instead of all the variables. Each selection produces an independent tree. Bootstrap aggregation, more commonly known as bagging, is the common procedure of random forests, which splits a training dataset into multiple data sets derived from bootstrapping. The results are contrasted against a validation test, from the observations (about one-third) not used for the training dataset. These observations are referred to as out-of-bag (OOB) observations. RF produce estimates on how many iterations are needed to minimize the OOB error. After selecting a number of trees, the algorithm averages the results and produces a robust classification method, which avoids overfitting of results to data, as is more common in standard decision and regression trees. Here, forests were built using 500 trees. For the present study, the “randomForest” and the “caret” R libraries were used. The final value used for the selected model was mtry = 5.

### Mixture Discriminant Analysis (MDA)

Initially conceived as an extension of LDA (Linear Discriminant Analysis), MDA is built upon class-specific distributions combined into a single multivariate distribution. This is done by creating a per-class mixture, as described by Kuhn and Johnson^32^. This consists of separating the class-specific means from the class-specific covariance structure. Otherwise described, each class has different means but the complete-class data set has the same covariance. These are sub-classes of the data. They are spatially modelled once it is specified how many distributions should be used. The tuning parameter for these models is the number of distributions per class or sub-classes. The MDA algorithm integrates ridge- and lasso- penalties to determine feature selection. Here, the final model selected through the kappa parameter was composed of 11 sub-classes.

### Naive Bayes (NB)

Bayes’ Rule, as used in the NB algorithm, estimates probabilities of classes on observed predictors (i.e., probabilities of previous outcomes), resulting in dynamic estimates of posterior probabilities of classes. The conditional probability (i.e., the probability of observing specific predictor values in relation to data associated with specific classes) is used to model classification. NB assumes that all predictors are independent. Prior probabilities allow the decision of which class any case must be assigned to. If no prior estimates are provided, these are derived from the documented occurrence of classes within the training set and their relation to predictors’ properties. Predicted classes are created based on the largest class probabilities for each class as derived from the training set. NB uses a nonparametric density modelling process. Here, the “e1071” qne “klaR” R libraries were used. The tuning parameter was held constant at a value of 0 and the kappa parameter was used to select the optimal model.

### Partial Least Squares Discriminant Analysis (PLSDA)

This test classifies the criterion variable classes by identifying the predictor combinations that optimally separate classes. It is commonly used in situations where predictor reduction is necessary (such as in LDA based on PCA scores), but it is more efficient than these two-step data reduction methods. PLSDA finds latent variables (components) that maximize classification accuracy. Therefore, when data reduction is required for classification, PLSDA is preferred over PCA-LDA. In this test, the tuning parameter is the number of latent components to be retained in the final model. When the number of predictors is short compared to the number of cases, PLSDA can execute classification better than LDA: Predictor importance can be also identified. Here, the “plsr” function within the “pls” R libary was used. Model tuning was carried out with the “caret” R library. The number of components retained in the final model was ten.

### Decision Trees using the C5.0 algorithm (DTC5.0)

This algorithm implemented in decision trees has enabled this ML technique to reach a degree of accuracy comparable to far more complex ML methods such as neural networks or support vector machines. The procedure is similar to that for simple decision trees. These operate through recursive partitioning of data. Decision trees can produce models whose performance can be improved with meta-learning methods. One of these methods is k-fold cross-validation. This consists of dividing the data set into k-subsets and the holdout method (original data set divided into training and testing sets) is repeated k times. The variance of the resulting estimate decreases as the k increases. The data thus randomly divided into k different sets produce results that are eventually averaged over. The standard number of trials is 10, but here, the models required 25 trials for the complete set and 60 for the partial set. A 10-fold cross validation method was adopted in both cases. For the present analysis, the “C50” and the “caret” R libraries were used. The numbers of rules adopted were 36 and 32 respectively and the number of trees used were <100 before convergence was reached.

## Electronic supplementary material

Supplementary Information
