# Supplementary material for: Distinguishing butchery cut marks from crocodile bite marks through machine learning methods
Source: Sci Rep. 2018 Apr 10;8:5786. doi: 10.1038/s41598-018-24071-1 (PMC5893542; doi:10.1038/s41598-018-24071-1)
Supplement: Supplementary file 1 — Supplementary Information [file 41598_2018_24071_MOESM1_ESM.docx]

**SUPPLEMENTARY INFORMATION**

**Distinguishing butchery cut marks from crocodile bite marks through machine learning methods**

Manuel Domínguez-Rodrigo^1,2*^, Enrique Baquedano^1,3^

^1^Institute of Evolution in Africa (IDEA), University of Alcalá de Henares, Covarrubias 36, 28010 Madrid, Spain.

^2^Department of Prehistory, Complutense University, 28040 Madrid, Spain

^3^Regional Archaeology Museum, Alcalá de Henares, Plaza de las Bernardas s/n, 28801 Madrid, Spain

*manueldr@ghis.ucm.es

1. **Variables used in the ML analysis.**

*Table S1. Definition of each of the variables used for the ML analysis.*

| 1. **Trajectory of the groove**. Marks can show a straight trajectory (1a), a curvy one (1b), a sinuous one (1c) and a variable one (1d). The latter involves changes in the trajectory direction more than twice. This categorization applies to most of the outline of the mark, excluding the presence of barbs at the end of the mark. Butchery marks are commonly straight grooves. In some cases, the abrasive marks created by sediment grains show a somewhat sinuous trajectory in part of the groove due to the rolling of the grain and the use of different edges of the grain for abrading the bone surface. Some apparently straight trampling marks, when observed under magnification, show trajectories that are not perfectly straight but are rather somewhat wavy. The movement of bones during the tight grasping by the jaw makes some crocodile tooth scores change direction. |
| --- |
| 1. **Presence (2a) or absence (2b) of a barb**. In some butchery marks, a barb can be observed at the end of the straight groove, defined as a shallower end of the groove slightly curved to the side in the form of an open hook. Testing how frequent this feature is in cut marks and in trampling marks can be potentially important, since it has also been observed in the latter. |
| 1. **Orientation of the mark**, relative to the axis of the bone. The orientation can be parallel (3a), perpendicular (3b) or oblique (3c) to the axis of the bone. Trampling marks, in theory, should show no preference in orientation, whereas butchery marks should be more frequently oriented obliquely or perpendicularly to the axis of the bone. |
| 1. **Shape of the groove**. The shapes used are: narrow V-shape (4a), wide V-shape (\_/) (4b) and U-shaped (4c). The wide V-shaped section is understood as either V- or \_/-shaped but either almost as deep as it is wide, or deeper than it is wide; the latter is understood as an open groove with a broader horizontal base and, therefore, substantially wider (by an order of magnitude >X2) than deeper. |
| 1. **Number of conspicuous grooves** per bone specimen. It has been mentioned that cut marks occur in lower numbers per specimen than trampling marks. |
| 1. **Symmetry of the groove**: the section and both sides of the groove can be symmetrical (6a) or asymmetrical (6b). The tilting of a stone tool during use can create asymmetrical grooves, and so can certain sediment particles during bone abrasion. |
| 1. **Shoulder effect** and associated shallower striae. Here we define the term as the striae occurring in association with the main groove in a distance not farther than 0.2 mm from the edge of the groove. For this type of analysis, a binocular lens with measuring capability is preferred. These striae frequently are shallow striations occurring parallel to or intersecting with the sides of the groove. They can be present (7a) or absent (7b) and have been documented in trampling marks, cut marks and crocodile bite marks |
| 1. **Presence of flaking** on the shoulders of the groove. The presence (over more [8a] or less [8b] than one-third of the trajectory of one or two shoulders of the groove) or absence (8c) of flaking on the shoulders of the groove can be related to the morphology of the abrasive agent: the bigger and less straight the edge of this agent the bigger the chance that such flaking would appear. Flaking here is defined as not random occurrence of a flaking dent such as those produced in isolated Hertzian cones, but as a continuous series of exfoliation of the shoulder edge, which can occur on part of the trajectory of the shoulder or on most of it. |
| 1. **Extent of the flaking** of the shoulder. The extent of the flaking could also be indicative of the abrasive agent. The category of the flaking can be defined as long (9a) when it occurs over a minimum of one-third of the trajectory of the groove and short (9b) when it is shorter than one-third. Approximate estimates can be made with hand lenses. |
| 1. **Striae overlapping** or running across the main groove with an oblique angle: present (10a) or absent (10b). These striae are frequently shallow in trampling marks. |
| 1. **Internal microstriations**. Defined as present (11a) or absent (11b) and observable under 40x. |
| 1. **Microstriation trajectory**. Defined as continuous (12a) when it extends along all the trajectory of the groove or discontinuous (12b) when the microstriations are interrupted at more than one instance inside the groove. A tool is more likely to create continuous microstriations given that it creates uniform friction in its contact with bone. A trampling mark is more likely to created discontinuous microstriations if friction forces the sediment particle to move inside the groove. This is also documented in crocodile bite marks given the movements of teeth during the grasping of the bone. |
| 1. **Shape of microstriation trajectory**. Defined as straight (13c) or irregular (13b), the latter including any other shape (curved, sinuous, combination of forms). |
| 1. **Location of microstriations**. On the walls of the groove (14a), on the bottom (14b) or on both (14c). |
| 1. **Length of the main groove** (in mm). |
| 1. **Associated shallow striae** (micro-abrasion) on the bone specimen away from the main groove (contextual approach): absence (16a) or presence (16b). We only tally presence or absence when identified under magnification <40x since in our experience we have documented that such striae can be detected most of the time under this magnification. These striae are very shallow and often not easy to perceive if one is not looking for them explicitly. They are caused by the sediment grains of sandy gritty soils which are part of the substrate where trampling takes place. |
| 1. **Associated tooth pits on mid-shafts.** Experiments show that carnivore tooth pits on mid-shaft portions are very uncommon in anthropogenic assemblages where carcass exploitation is primarily carried out by humans. In contrast, in crocodile-modified assemblages, they occur with some regularity. |

Variables 5,10, 16 and 17 are extrinsic (i.e., contextual). The other variables are intrinsic (i.e., structural).

1. **Differences between butchery BSMs and crocodile BSMs and classification of the Middle Awash fossil BSMs.**

Sahle et al. [1] claim that no differences were found between butchery and crocodile bite marks using 3D information derived from the confocal profilometer microscope. Here, we use their supplementary Table 1, containing their profilometer information to show that differences between both experimental sets are profound and statistically significant. We also use their profilometer information to show that three of their Middle Awash fossil bones bear marks that most closely resemble butchery marks and another one remains ambiguous, although it clusters in the periphery of the crocodile-modified sample.

Initially, a hierarchical classification (HC) of the principal components of a factorial analysis was carried out on the whole sample. Variables are mathematically heterogenous and previously to being analysed, they were scaled and centered. Then a PCA was carried out and the loading scores were used for a hierarchical classification. This was done with the PCA and HCPC functions of the R “FactomineR” library. This showed that there was an outstanding outlier (Mak-VP-1-3) (Figure S1). This could bias the comparative analysis and for this reason, in subsequent analyses, it was removed.

_
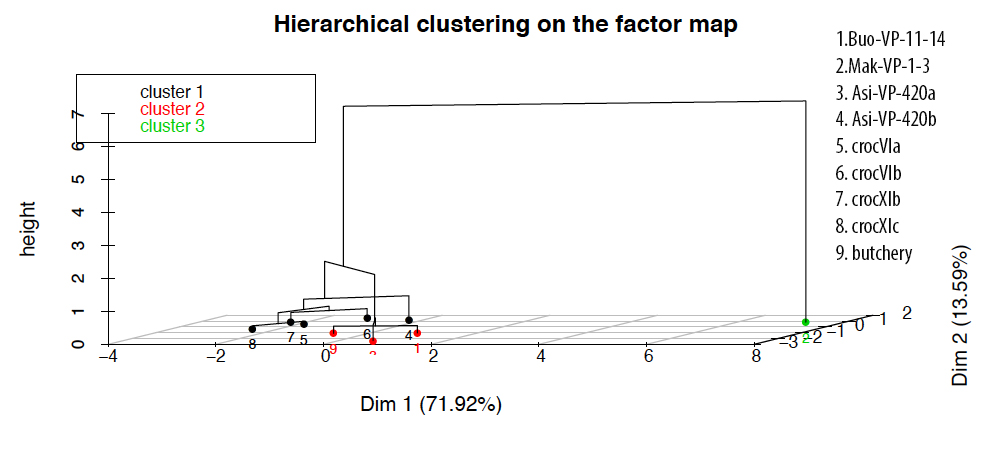
_

*Figure S1. HC analysis on PCA scores for Shale et al´s Table 1.*

The exploratory HC-factor analysis showed that not all the variables were equally important for the two-component solution. This stressed the need of finding the most diagnostic variables, which could account for an even bigger portion for the inter-group sample variance. Instead of continuing with a PCA for variable trimming, it was decided to use a machine learning (ML) approach. A random forest was used for this purpose (see description in Method). The random forest (RF) was used on a slightly bootstrapped sample (n=100) of the experimental data set of Sahle et al.´s [1] Table 1, and excluding the fossil bones. This bootstrapped sample yielded a classification of crocodile BSM and butchery BSM with an accuracy of 100% as shown in confusion matrices and an out-of-bag error (OOB) of 0%. This RF analysis showed that variables were heterogeneously important for correct classification between both experimental sets (Figure S2). The mean accuracy index and the Gini index showed that a set of variables were more important for inter-group variance. In Figure S3, the first 8 variables selected by the Gini index (supported by the mean accuracy index) were selected for posterior analyses, given the separation between “surface” and “area” in the Gini index.


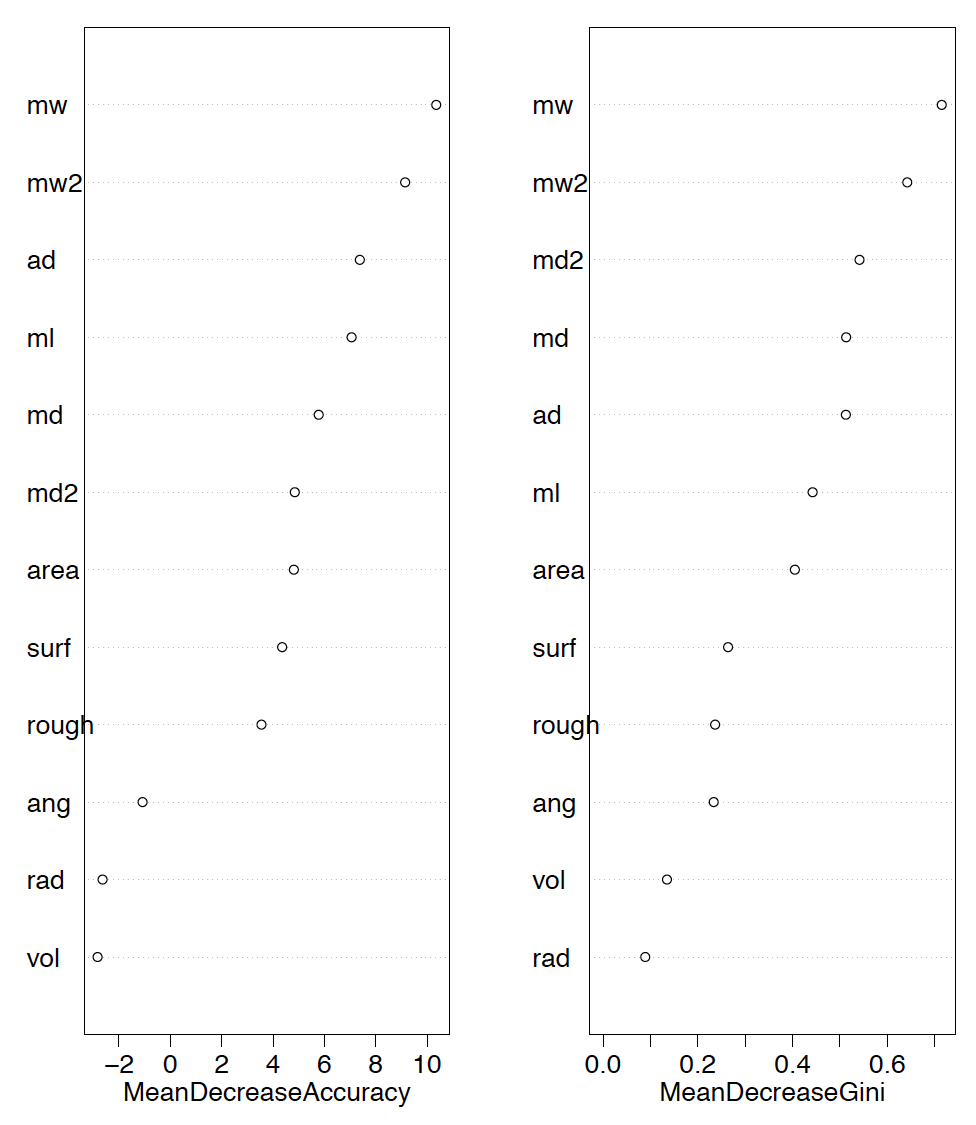


*Figure S2. Importance of variables for correct classification of the experimental samples in Sahle et al.´s [1] Table 1. See key for variables in Appendix.*

The subsample of variables selected by the RF was used in additional HC analyses, which showed an increase in the accuracy of classification and association of BSM (Figure S3).


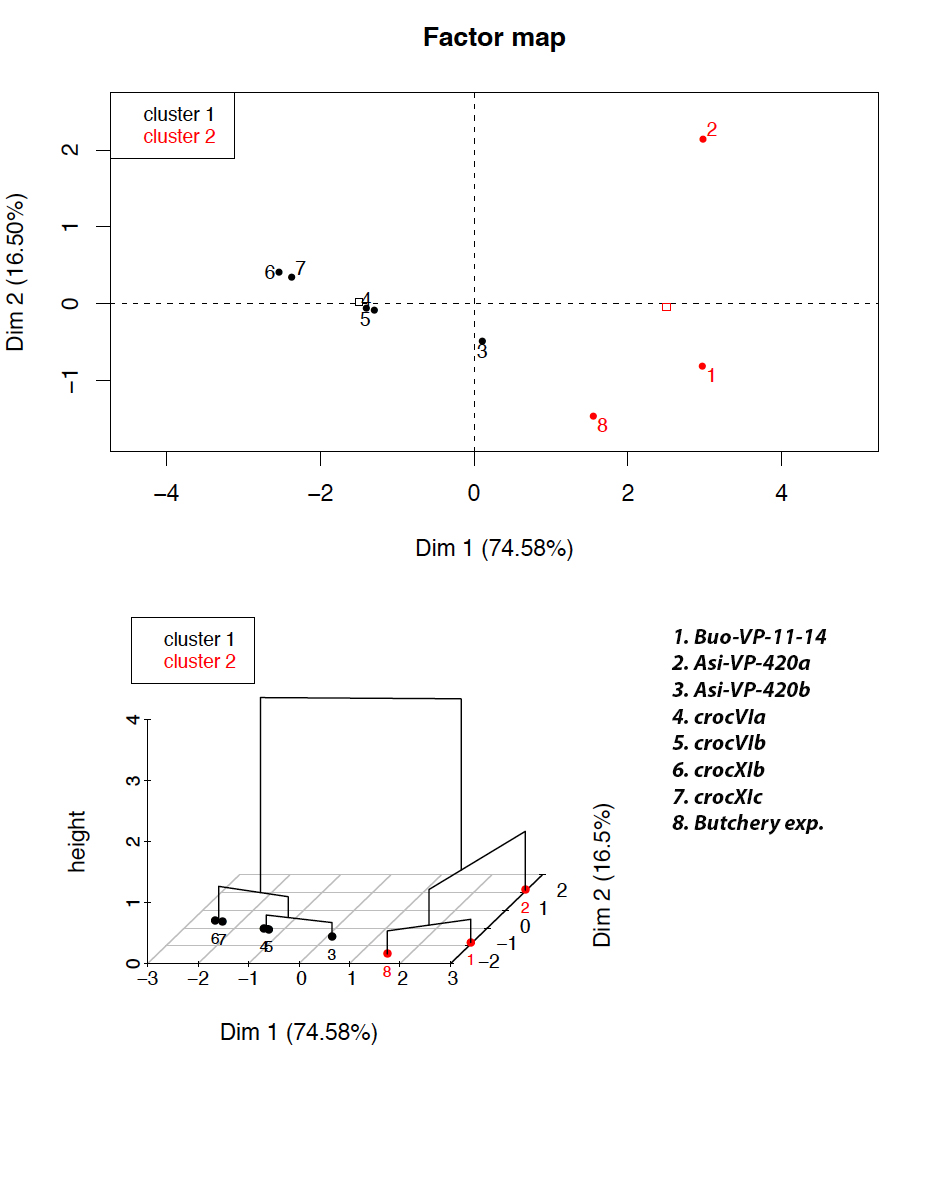


*Figure S3. HC analysis on PCA scores for Shale et al´s Table 1, after removing the outlier from Figure S1. The HC analysis identifies two clusters of data. The crocodile BSM are clearly separated from the rest of the sample.*

A K-means (KM) partitioning algorithm was also used to identify clusters in a multidimensional Euclidean space. This semi-unsupervised algorithm was selected for its flexibility and its high efficiency at dividing data into useful clusters. The classifying procedure creates *k* clusters. To select the optimal number of clusters, the Calinski index (F-statistic comparing inter- and intra-group SS of the partition) was selected in combination with a bootstrapping clustering method with a CBI (Cluster Bootstrap Interface) of k-means as described in [2]. Several (n=50) bootstrap samples were drawn from the original sample and the number of clusters is derived by optimization of bootstrapped pairs. We compared the number of clusters selected by both algorithms. Both coincided in their recommendations. Figure S4 shows how the divergences among all the cases and groups in Sahle et al´s Table 1 are so profound that the recommendation was for a minimum of 8 groups.


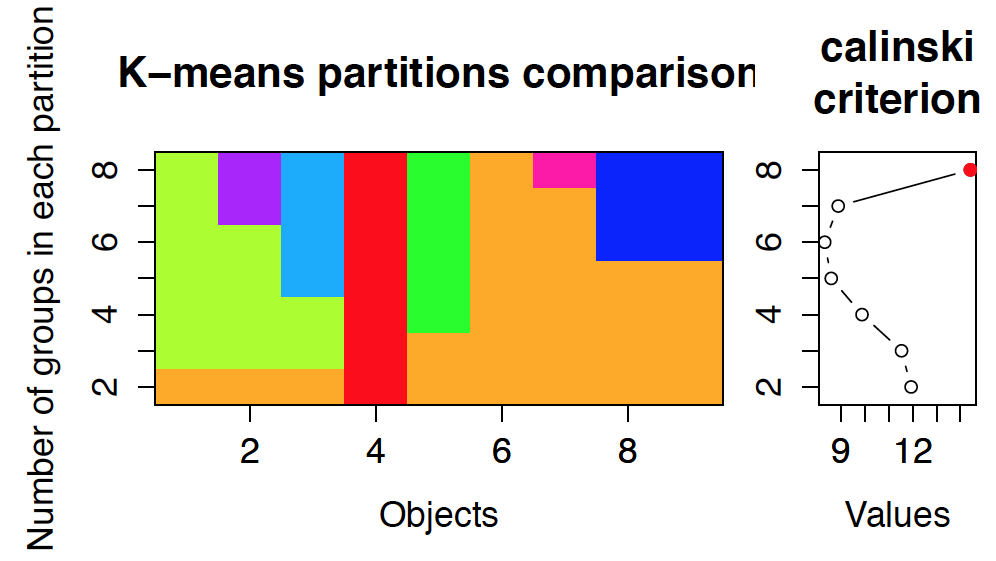


Figure S4. K-clustering recommendation of Sahle et al.´s Table 1 using the Calinkski criterion. This was carried out with the “cascadeKM” function of the “vegan” R library.

This within-sample variance indicates that: a) there is a high degree of variability in the fossil BSM and they should not be interpreted as caused by a single or dual agency; b) the range of variation in the fossil BSM sample is also wide, showing that the profilometer information may suggest a variety of agents not contemplated in the crocodile-butchery dichotomy.

We adopted the most conservative approach and treated the whole sample as a single group to document its distribution on the Euclidean space. A KM analysis showed a bi-dimensional solution that accounted for 89.89% of all sample variance. In this KM solution, it can be clearly seen that three of the Middle Awash fossils cluster with the single butchery BSM experiment. Only one fossil bone shows marks that are intermediate between the butchery and crocodile samples. None is clearly associated with crocodile BSM. This clearly shows that the profilometer 3D data are showing a clear separation between the crocodile BSM experimental samples and the rest (Figure S5).


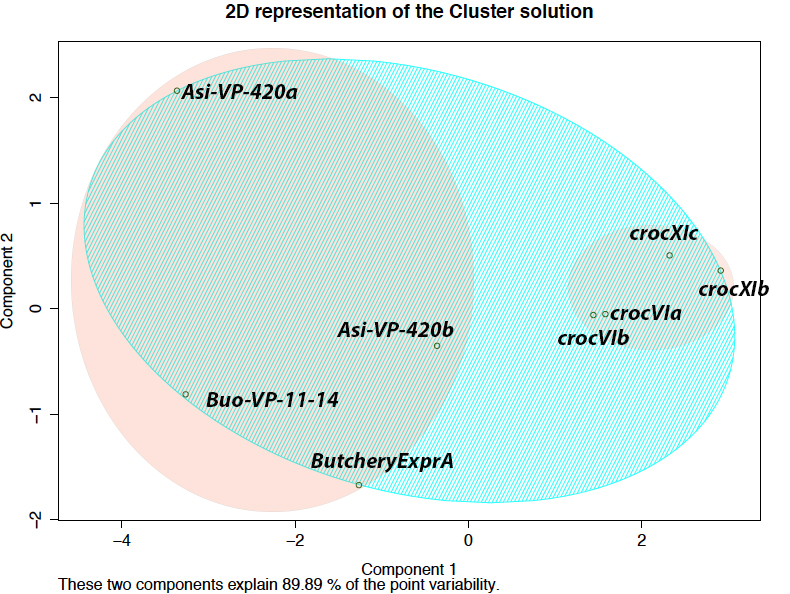


*Figure S5. KM analysis and selection of two clear groups (human butchery and crocodile bite marks), with the Middle Awash fossil BSM clustering closer to the experimental butchery BSM.*

With almost 90% of inter-group variance explained, the KM analysis was clearly showing that none of the Middle Awash BSM resembled the experimental crocodile BSM set. Three of them clustered with the values for the experimental butchery BSM.

As a further support for this, we used the ML RF algorithm on the complete data set (including all the original variables displayed in Sahle et al.´s [1] Table 1) trained on the crocodile bite marks and the experimental butchery marks for attempting the classification of the Middle Awash BSMs. This yielded the results showed in Table S2. Three of the fossils BSM sets were classified as butchery marks with high probabilities and the fourth one was classified as crocodile BSMs with somewhat lower probability.

*Table S2. Classification and probabilities yielded by the RF algorithm on each BSM data set from the Middle Awash.*

|  | classification | Probability butch | Probability croc |
| --- | --- | --- | --- |
| Bou-VP-11-14 | Butchery BSM | 0.791 | 0.209 |
| Mak-VP-1-3 | Butchery BSM | 0.791 | 0.209 |
| Asi-VP-420a | Butchery BSM | 0.780 | 0.220 |
| Asi-VP-420b | Crocodile BSM | 0.286 | 0.714 |

If using only the set of variables selected by the RF algorithm as the important ones (se Figure S2), the RF yields a more reassuring result. The probabilities of classification are 100% as butchery marks for the first three fossil BSM specimens and ambiguous crocodile for the fourth one (Table S3).

*Table S3. Classification and probabilities yielded by the RF algorithm on each BSM data set from the Middle Awash on the selected variable set, as indicated by the GINI and Mean Decrease Accuracy indeces.*

|  | classification | Probability butch | Probability croc |
| --- | --- | --- | --- |
| Bou-VP-11-14 | Butchery BSM | 1.000 | 0.000 |
| Mak-VP-1-3 | Butchery BSM | 1.000 | 0.000 |
| Asi-VP-420a | Butchery BSM | 1.000 | 0.000 |
| Asi-VP-420b | Crocodile BSM | 0.424 | 0.576 |

The conclusions from the data set provided by the profilometer in Sahle et al. [1] indicate that:

1. Crocodile bite marks are clearly different from butchery marks.
2. None of the selected fossil BSM from Middle Awash can unambiguously be mistaken with crocodile-inflicted marks.
3. Most of the selected Middle Awash BSM are much more similar to experimental butchery marks.
4. Formal descriptive use of univariate or bivariate descriptions of BSM lead to an equifinality that does not exist when multivariate approaches are used, even if focused only on profilometer metric data.

Despite this statistically similarity between most of the Middle Awash BSMs and the butchery experimental BSM, it should not be concluded that those fossils represent some of the earliest traces of hominin butchery. First, the RF algorithm was only trained on the limited data provided by Sahle et al. [1]. This limits the algorithm knowledge of BSMs to just a few modifications from two agents (humans and crocodiles). The RF algorithm needs be to trained on a larger array of other taphonomic agents (including a larger range of human butchery behaviors and associated tools) to heuristically conclude that the Middle Awash BSMs are indeed butchery marks. Secondly, the KM and HC analyses showed that the Middle Awash fossils BSM did not cluster closely with the experimental butchery mark set, but shared with it their distance from crocodile marks and, in contrast, they diverged substantially in their location within the Euclidean space (Figures S3 and S5). This is especially relevant for the Mak-VP-1-3 that stood out as a remarkable outlier (Figure S1). This indicates that the Middle Awash BSMs probably represent a much larger number of agents and mark types than the unfortunately limited butchery-crocodile dichotomy defended by Sahle et al [1]. This probably includes abrasion marks caused by trampling in coarse-grained sediments or trampling, as well as other non-documented factors.

It is, however, interesting to note, in support of the statistical analyses here presented, that the Middle Awash specimen Bou-VP-11-14 clusters so close with the butchery experimental sample (Figures S3 and S5). According to Sahle et al.[1], this specimen is “difficult to interpret”. It bears conchoidal fractures that “cannot be distinguished from hammerstone percussion”. Typical “crocodile bisected pits are absent”. “Irregular pit with internal striations are present” and these are typical of hammerstone percussion marks. Although Sahle et al. feel now indecisive as to the attribution of the BSMs of this specimen to specific agent(s), this ungulate tibia made a compelling example of hominin butchery [3]. Some of the linear grooves are similar to experimental chop marks made with modified hammerstones [4]. The profilometric properties of this specimen´s BSM support a hominin agency over any other considered alternative. This requires further work, but the fact that none of the “more than 40 separate modifications” of this specimen can be safely attributed to crocodiles detracts from interpreting this specimen as crocodile made, given that in our experimental collection, *all* the crocodile-modified specimens bearing more than 6 BSM show at least one typical bisected mark that can unambiguously be identified as crocodile-made.

The present analysis reinforces an abundant literature stressing that the old univariate description of butchery cut marks as linear V-shaped marks with internal striae (resurrected by Sahle et al.[1]) is useless for taphonomic research because it methodologically *creates* equifinality. This work also shows that simple multivariate approaches to metric data (complementing the multivariate categorical data in the main text) clearly reveals that most crocodile bite marks and butchery marks should not be mistaken. Finally, statistical treatment of the metric data provided by Sahle et al.[1] also shows that their interpretation of crocodile modifications in most of the Middle Awash selected fossils specimens is unsupported and can be rejected using their own profilometric data. The ensuing speculation by these authors about the abundant presence and high impact of crocodiles on Plio-Pleistocene paleolandscape and hominin lives lies, therefore, in an epistemological vacuum.

**References**

[1] Sahle et al. (2017). Hominid butchers and biting crocodiles in the African Plio-Pleistocene. Proc. Nat. Acad. of Scienc.

[2] Fang, Y., Wang, J. (2012). Selection of the number of clusters via the bootstrap method. Comp. Stat. Data Anal. 56: 468-477.

[3] De Henzelin J. et al. (1999). Environment and behavior of 2.5-million-year-old Bouri hominids. Science 284: 625-629.

[4] Galan AB et al. (2009). A new experimental study on percussion marks and notches and their bearing on the interpretation of hammerstone-broken faunal assemblages J. Arch. Sci. 36: 776-784.

**Appendix**

Key to the variables used in the present analysis:

| Surface area (3D studiables) | surf |
| --- | --- |
| Volume (3D studiables) | vol |
| Maximum depth (3D studiables) | md |
| Mean depth (3D studiables) | ad |
| Maximum length (deepest profile) | ml |
| Maximum width (deepest profile) | mw |
| Maximum depth (deepest profile) | md2 |
| Area (deepest profile) | area |
| Maximum width (deepest profile) | mw2 |
| Roughness | rough |
| Angle | ang |
| Radius | rad |

These variables and the associated data are from Sahle et al´s [1] Table 1.
